# Deregulated Immune Pathway Associated with Palbociclib Resistance in Preclinical Breast Cancer Models: Integrative Genomics and Transcriptomics

**DOI:** 10.3390/genes12020159

**Published:** 2021-01-25

**Authors:** Kamal Pandey, Eunbyeol Lee, Nahee Park, Jin Hur, Young Bin Cho, Nar Bahadur Katuwal, Seung Ki Kim, Seung Ah Lee, Isaac Kim, Hee Jung An, Sohyun Hwang, Yong Wha Moon

**Affiliations:** 1 Hematology and Oncology, Department of Internal Medicine, CHA Bundang Medical Center, CHA University, Seongnam 13496, Korea; pkamal@chauniv.ac.kr (K.P.); skgml0413@naver.com (N.P.); hurjinz@naver.com (J.H.); ybyoungbin@naver.com (Y.B.C.); 2 Department of Biomedical Science, The Graduate School, CHA University, Seongnam 13496, Korea; byeol44@naver.com (E.L.); narbahadurkatwal@gmail.com (N.B.K.); 3 Department of Surgery, CHA Bundang Medical Center, CHA University, Seongnam 13496, Korea; mdsky@cha.ac.kr (S.K.K.); mdseungah@chamc.co.kr (S.A.L.); isaac24@cha.ac.kr (I.K.); 4 Department of Pathology, CHA Bundang Medical Center, CHA University, Seongnam 13496, Korea; hjahn@cha.ac.kr

**Keywords:** hormone receptor-positive breast cancer, CDK4/6, drug resistance, genomics, transcriptomics, immune pathway

## Abstract

Recently, cyclin-dependent kinase (CDK) 4/6 inhibitors have been widely used to treat advanced hormone receptor-positive breast cancer. Despite promising clinical outcomes, almost all patients eventually acquire resistance to CDK4/6 inhibitors. Here, we screened genes associated with palbociclib resistance through genomics and transcriptomics in preclinical breast cancer models. Palbociclib-resistant cells were generated by exposing hormone receptor-positive breast cancer cell lines to palbociclib. Whole-exome sequencing (WES) and a mRNA microarray were performed to compare the genomic and transcriptomic landscape between both palbociclib-sensitive and resistant cells. Microarray analysis revealed 651 differentially expressed genes (DEGs), while WES revealed 107 clinically significant mutated genes. Furthermore, pathway analysis of both DEGs and mutated genes revealed immune pathway deregulation in palbociclib-resistant cells. Notably, DEG annotation revealed activation of type I interferon pathway, activation of immune checkpoint inhibitory pathway, and suppression of immune checkpoint stimulatory pathway in palbociclib-resistant cells. Moreover, mutations in *NCOR1, MUC4*, and *MUC16* genes found in palbociclib-resistant cells were annotated to be related to the immune pathway. In conclusion, our genomics and transcriptomics analysis using preclinical model, revealed that deregulated immune pathway is an additional mechanism of CDK4/6 inhibitor resistance besides the activation of cyclin E-CDK2 pathway and loss of RB, etc. Further studies are warranted to evaluate whether immune pathways may be a therapeutic target to overcome CDK4/6 inhibitor resistance.

## 1. Introduction

Breast cancer, the most common type of cancer and one of the leading causes of mortality among women worldwide [1], can be classified into three subtypes, hormone receptor (HR)-positive, human epidermal growth factor receptor (HER) 2-positive, and triple negative breast cancer. Among the three, HR-positive breast cancer has been the most common, constituting approximately 70% of all breast cancer subtypes [2].

Cyclin-dependent kinase (CDK) 4/6 inhibitors in combination with endocrine therapy had been approved by the US Food and Drug Administration as a first-or second-line treatment of HR-positive/ HER2-negative breast cancer [3]. CDK4/6 inhibitors prevent retinoblastoma (RB) protein phosphorylation and eventually restrict G1 to S phase cell progression [4]. Despite evidence of clinical benefit, concerns regarding CDK4/6 inhibitor resistance have been emerging [5,6,7]. Although several mechanisms associated with CDK4/6 inhibitor resistance, including RB loss [8], cyclin E overexpression [9], FGFR amplification [10], PTEN loss [11], and MDM2 amplification [12], have been investigated, no clinical biomarker and validated strategies for overcoming CDK4/6 inhibitor resistance are as yet available.

Immune pathways, such as immune checkpoints, have been considered imperative in cancer progression and drug resistance [13]. Various immunomodulatory cytokines, such as IL4, IL6, and TGF-β, produced by immune cells within the tumor microenvironment promote tumor growth and progression [14,15]. Consequently, immune checkpoint inhibitors have gained attention for being one of the most promising types of immunotherapy [16]. In addition, various combinatorial strategies are currently being implemented to enhance the efficacy of immune checkpoint inhibitors [17]. For instance, studies have shown that combining immunotherapy with various chemotherapies and targeted therapies enhanced immune response and promoted enhanced checkpoint inhibitor efficacy by turning immunologically cold tumors into hot ones [17]. Furthermore, other studies have demonstrated the mechanistic association between CDK4/6 inhibition and immune response [18,19]. In more detail, one study reported that CDK4/6 inhibition promoted T cell activation via enhancement of IL2 secretion and modulation of nuclear factor of activated T-cells activity [18]. Another study also demonstrated that CDK4/6 inhibitors altered the immune microenvironment by stimulating the production of type III interferon (IFN) and suppressing regulatory T cell proliferation [19]. Despite the enormous clinical benefits of CDK4/6 inhibitors, acquired resistance has continued to be a concern, with various studies regarding the mechanism of CDK4/6 inhibitor resistance being conducted as mentioned earlier. However, only a few studies have investigated the role of immune pathways in CDK4/6 inhibitor resistance.

Therefore, the present study generated palbociclib-resistant preclinical breast cancer models using HR-positive breast cancer cells and investigated mechanisms associated with palbociclib resistance through integrative genomics and transcriptomics focusing mainly on immune pathways.

## 2. Materials and Methods

### 2.1. Cell Culture and Resistant Cell Line Establishment

**Figure 1:**
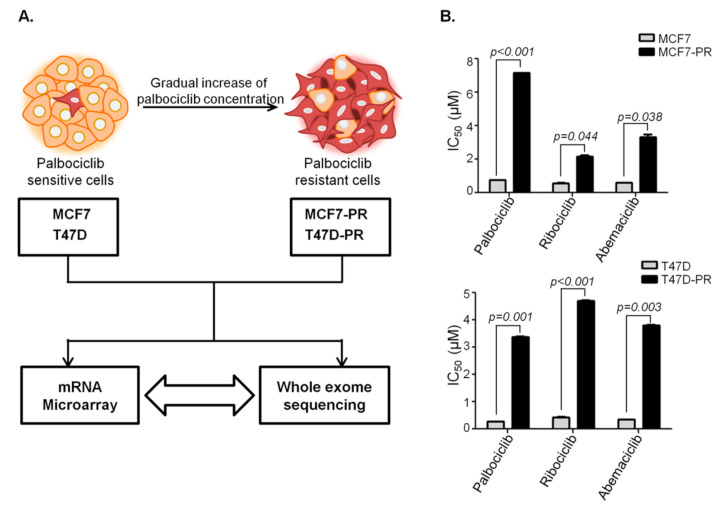
Schematic design of the current study. Generation of palbociclib-resistant cells. (**A**) Palbociclib-resistant HR-positive breast cancer cells, indicated as MCF7-PR and T47D-PR, were generated by gradually exposing MCF7 and T47D cells to increasing concentrations of palbociclib. (**B**) The IC50 of palbociclib in MCF7-PR and T47D-PR cells increased by around 10-fold. MCF7-PR and T47D-PR cells were cross-resistant to ribociclib and abemaciclib. *P* values were calculated by Student’s *t*-test. Data are presented as means ± standard deviation of triplicate experiments. Palbociclib-resistant cells and their sensitive counterparts were compared using microarray analyses and whole-exome sequencing.

HR-positive cells MCF7 and T47D were obtained from The American Type Culture Collection (Manassas, VA, USA), subsequently cultured in RPMI 1640 medium (Welgene Inc., Daegu, Korea) supplemented with 10% heat-inactivated fetal bovine serum (Welgene Inc., Daegu, Korea) and 1% 100× penicillin/streptomycin solution (Welgene Inc., Daegu, Korea). The CDK4/6 inhibitor palbociclib was provided by Pfizer Inc. (North Peapack, NJ 07977, USA). Palbociclib-resistant cells, indicated as MCF7-PR and T47D-PR, were generated from MCF7 and T47D cells by treating with palbociclib for approximately 9 months in a stepwise dose escalating fashion as described previously [20] (Figure 1A). Resistant cells gained a more than 10-fold higher half-maximal inhibitory concentration (IC_50_) than their parental counterparts: 7.15 μM in MCF7-PR vs. 0.75 μM in MCF7 and 3.37 μM in T47D-PR vs. 0.26 μM in T47D-PR. Palbociclib-resistant cells also showed cross resistance to ribociclib and abemaciclib (Figure 1B).

### 2.2. Gene Expression via Microarray Analyses

Microarray analysis of palbociclib-sensitive cells and resistant cells was performed using the Affymetrix GeneChip Human 2.0 ST Array (Affymetrix, Cleveland, OH, USA). cDNAs of each sample were synthesized using the GeneChip WT (Whole Transcript) Amplification Kit according to the manufacturer’s protocol. Thereafter, cDNAs were used for expression profiling using GeneChip^®^ Hybridization, washed, and stained on a GeneChip Fluidics Station 450. The probe array was scanned using the GCS3000 Scanner (Affymetrix, Cleveland, OH, USA) and analyzed using the Affymetrix^®^ GeneChip™ Command Console software. Data preprocessing, such as background correction, summarization, and normalization, were performed through RMA analysis in Affymetrix Power Tools.

### 2.3. Whole Exome Sequencing (WES)

To generate standard exome capture libraries, we used the Agilent SureSelect Target Enrichment protocol for the Illumina Paired-End Sequencing Library (Version C2, December, 2018) together with a 1-µg input of genomic DNA. In all cases, the SureSelect Human All Exon V6 probe set was used. DNA quantity and quality were measured using PicoGreen and Nanodrop. Fragmentation of 1-µg genomic DNA was performed using Adaptive Focused Acoustics technology (Covaris, Woburn, MA, USA). The fragmented DNA was repaired (i.e., an “A” was ligated to the 3′ end), and Agilent adapters were then ligated to the fragments. Once ligation had been assessed, the adapter-ligated product was polymerase chain reaction (PCR) amplified. The quantity and quality of the final purified product was then determined using the TapeStation DNA screentape (Agilent, Santa Clara, CA, USA). For exome capture, 250 ng of DNA library was mixed with hybridization buffers, blocking mixes, RNase block, and 5 µL of SureSelect all exon capture library according to the standard Agilent SureSelect Target Enrichment protocol. Hybridization to the capture baits was conducted at 65 °C using a heated thermal cycler lid set at 105 °C for 24 h on a PCR machine. The captured DNA was then amplified, after which the quantity and quality of the final purified product were determined using qPCR (according to the qPCR Quantification Protocol Guide) and TapeStation DNA screentape (Agilent), respectively. Finally, we sequenced using the NovaSeq platform (Illumina, San Diego, CA, USA).

Sequences were aligned using BWA-0.7.12 [21] based on Genome Reference Consortium build 37 (GRCh37). After duplicate reads were removed using Picard-tools-1.130, mutations were identified using the Genome Analysis Toolkit v3.4.0 following best practice guidelines [22]. Mutation annotation was performed using four public databases (i.e., 1000 Genomes Phase 3 [23], dbSNP 142, ESP (ESP6500SI V2), and ClinVar [24] (downloaded May 2015)) and one computational prediction method (i.e., SnpEff v4 [25]). The 1000 Genomes, dbSNP, and ESP databases were used for identifying common mutations, while clinical information regarding the pathogenicity of a mutation was determined using ClinVar. The SnpEff v2 tool was used to predict the effects of gene mutations, estimate the deleteriousness of the mutations, and classify them as “Modifier,” “Low,” “Moderate,” and “High.” For instance, large chromosomal deletions and duplications are mutations with High impact, gene duplication has Moderate impact, intron mutations have Modifier impact, and synonymous mutations have Low impact.

### 2.4. Pathway Analysis of Differentially Expressed Genes (DEGs)

To identify DEGs, we divided the fold change in mRNA expression of the palbociclib-resistant cells by that of sensitive cells (e.g., MCF7-PR/MCF7 or T47D-PR/T47D). GO Biology Process terms were used for pathway analysis. The gProfileR R package (0.7.0) was used to identify statistically enriched GO Biology Process terms in our DEGs. Statistical significance for pathway analysis was adjusted using the Benjamini–Hochberg method for multiple hypothesis testing correction.

### 2.5. Quantitative Real-Time PCR (qRT-PCR)

Total RNA was extracted using TRIzol (Life Technologies, Carlsbad, CA, USA) according to the manufacturer’s instructions. RNA was quantified and used to generate cDNA using the Takara PrimeScript 1st strand cDNA Synthesis Kit (Takara Bio Inc, Seoul, Korea). qRT-PCR was performed using a Power-up SYBR Green Master Mix (Thermo Fisher Scientific, Waltham, MA, USA), while mRNA detection was performed using an ABI Step One Real-time PCR System (Applied Biosystems, Foster City, CA, USA). The comparative CT method was used to determine the relative expression in each sample using β actin as normalized control. Primers were obtained from Macrogen (Macrogen, Inc., Seoul, Korea).

### 2.6. Cell-Mediated Cytotoxicity Asssay

MCF7-PR cell (1.5 × 10^5^ cells/well) were stained with carboxyfluorescein succinimidyl ester (CSFE) dye for 15 min and seeded in 6 well plates. Next day, Jurkat cells (1.5 × 10^5^ cells/well) were co-cultured with CFSE stained MCF7-PR cells by adding T cell stimulator, 10 μg/mL of phytohemagglutinin (PHA) and cells were treated with 25 μg/mL anti human PD-1 inhibitor (cat# BE0188, Clone: J116) for 48 h. The cells were harvested and stained with dead cell exclusion dye 7AAD. After 20 min of incubation, the cells were washed with PBS and then were analyzed by flow cytometry. Both CSFE and AAD stained cells are considered as an apoptotic cancer cells.

### 2.7. Identification of Driver or Pathogenic Mutations

To identify clinically important mutations, we initially removed common variants that had allele frequencies greater than 0.05 in the normal population and mutations with low quality (FILTER ≠ PASS). Thereafter, mutations in the coding region were selected. Lastly, pathogenic mutations were defined according to ClinVar, while mutation impact was predicted using SnpEff. Clinically important mutations, which are pathogenic or likely pathogenic in ClinVar, were identified, while High or Moderate impact mutations predicted by SnpEff were considered to be possibly pathogenic. A total of 203 clinically important mutations in 107 genes were identified in the T47D cells, while 312 mutations in 150 genes were identified in the MCF7 cells.

For the mutation dot plot, we focused on genes with greater significance than the aforementioned criteria. Firstly, we selected mutations with only High impact by removing mutations with Moderate impact. Secondly, we selected mutations in genes defined as a cancer gene by the COSMIC v87 cancer gene census (Tier 1 or 2) [26]. However, pathogenic mutations in ClinVar were not removed, although their genes were not included in COSMIC cancer gene census. Ultimately, 52 mutations in 30 genes were selected for the mutation dot plot.

### 2.8. Pathway Analysis for Genes with Driver Mutations by Visualizing Gene Ontology (GO)

The GO Biology Process terms were also used for pathway analysis of 107 and 150 genes with driver mutations in T47D and MCF7 cells, respectively. Statistically enriched biological process terms in genes with driver mutations were identified using the gProfileR R package (0.7.0). Based on the GO pathway analysis results, we then visualized enriched GO terms in our mutated genes using semantic similarity-based scatterplots in REduce and VIsulize Gene Ontology (REVIGO) [27]. To assist in interpretation, REVIGO summarizes and visualizes GO terms by finding a representative subset of terms using a simple clustering algorithm that relies on similarity measures between GO terms.

## 3. Results

### 3.1. DEG Analysis Revealing Deregulation of Immune Pathway in Palbociclib-Resistant Cells

**Figure 2:**
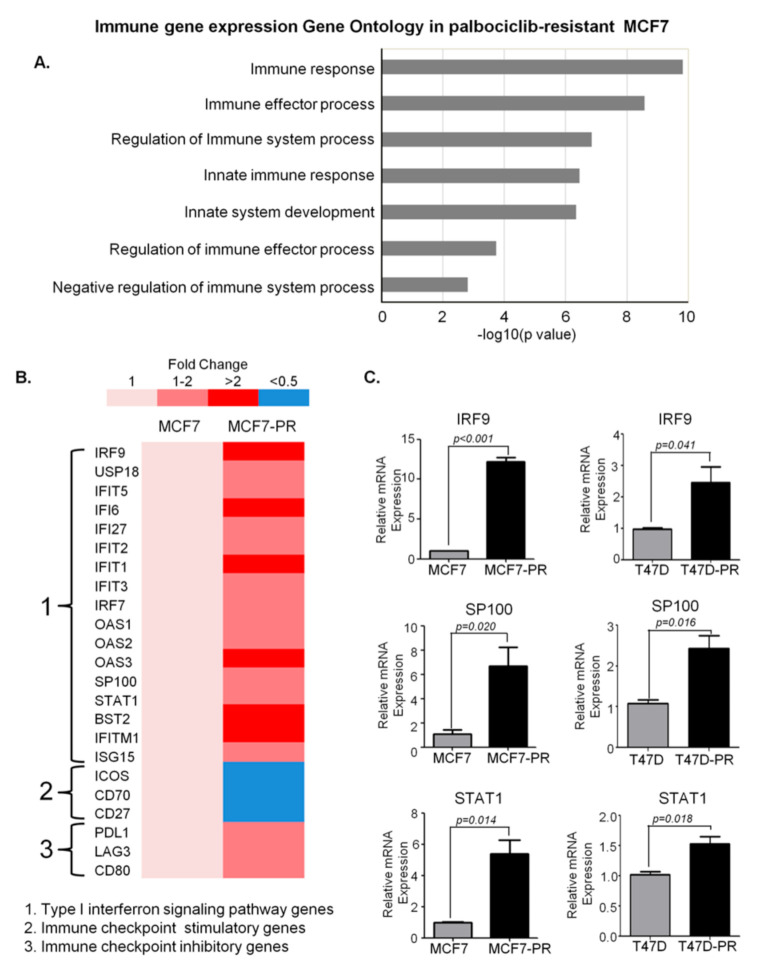
DEG analysis in the MCF7 cells revealing immune pathway deregulation in palbociclib-resistant cells. (**A**) To identify DEGs, the fold change in mRNA expression of the palbociclib-resistant cells was divided by that of the sensitive cells (e.g., MCF7-PR/MCF7 or T47D-PR/T47D). GO Biology Process terms were used for pathway analysis. The gProfileR R package (0.7.0) was used to identify statistically enriched GO Biology Process terms in our DEGs. The bar graph shows that GO biological process terms related to immune response were enriched in DEGs. The X axis indicates *p* values in the specified formula −log 10 (*p* value). The bar represents the statistical significance of each GO term. All GO terms in Figure 2 were statically significant (adjusted *p* value < 0.05). The gene list of each GO term overlapping with DEGs is provided in Table 1. (**B**) Panels of specific immune pathway genes are demonstrated. Type I IFN genes and immune checkpoint inhibitory/stimulatory genes were detailed in these panels to compare their expression levels between palbociclib-sensitive and resistant MCF7 cells. (**C**) Quantitative real-time PCR data demonstrate increased type I IFN signals. *P* values were calculated by Student’s *t*-test. Data are presented as means ± standard deviation of triplicate experiments.

To identify genes and pathways involved in the development of palbociclib resistance, microarray analysis was performed on both palbociclib-sensitive and resistant cells (Supplementary Figure S1A,B). The generation and confirmation of palbociclib resistant cells as shown in Figure 1A,B is explained in detailed in Materials and Methods section. Comparison of gene expression between sensitive T47D and resistant T47D-PR cells identified 210 DEGs (fold change >2 or <0.5), although no GO terms were enriched in all of them (Supplementary Figure S1B). However, a comparison between MCF7 and MCF7-PR cells identified 651 DEGs, with GO enrichment analysis revealing that 85 genes were involved in immune pathways (Figure 2A). Furthermore, all immune-related genes were classified into various categories according to fold changes as shown in Figure 2B. Given the previous reports demonstrating associations between the type I IFN pathway and endocrine resistance [28] and CDK4/6 inhibitor resistance [29] in the HR-positive breast cancer, a collection of type I IFN genes, such as *STAT1*, *IRF9*, and *SP100*, were analyzed in detail. Accordingly, type I IFN genes were found to have increased in MCF7-PR cells (Figure 2B), which was further validated by qRT-PCR (Figure 2C). Moreover, given the involvement of the immune checkpoint pathway in cancer progression and drug resistance [13,30], immune checkpoint inhibitory or stimulatory genes were also analyzed in detail. Notably, our results showed that while immune checkpoint inhibitory genes such as *PDL1, LAG3* and *CD89* were activated, stimulatory genes such as *ICOS, CD70* and *CD27* were suppressed in MCF7-PR cells (Figure 2B). The activation of PD-1/PD-L1 pathway in MCF7-PR cells was validated by cell mediated cytotoxicity assay. We co-cultured Jurkat cells with MCF7-PR cells and treated with PD-1 inhibitor. The addition of PD-1 inhibitor blocked the PD-1/PD-L1 pathway in MCF7-PR cells resulting in the increased activity of Jurkat cells to kill MCF7-PR cells (*p* = 0.002) (Supplementary Figure S1C).

### 3.2. Mutation Profiling Revealing Deregulation of Immune Pathway in Palbociclib-Resistant Cells

**Figure 3:**
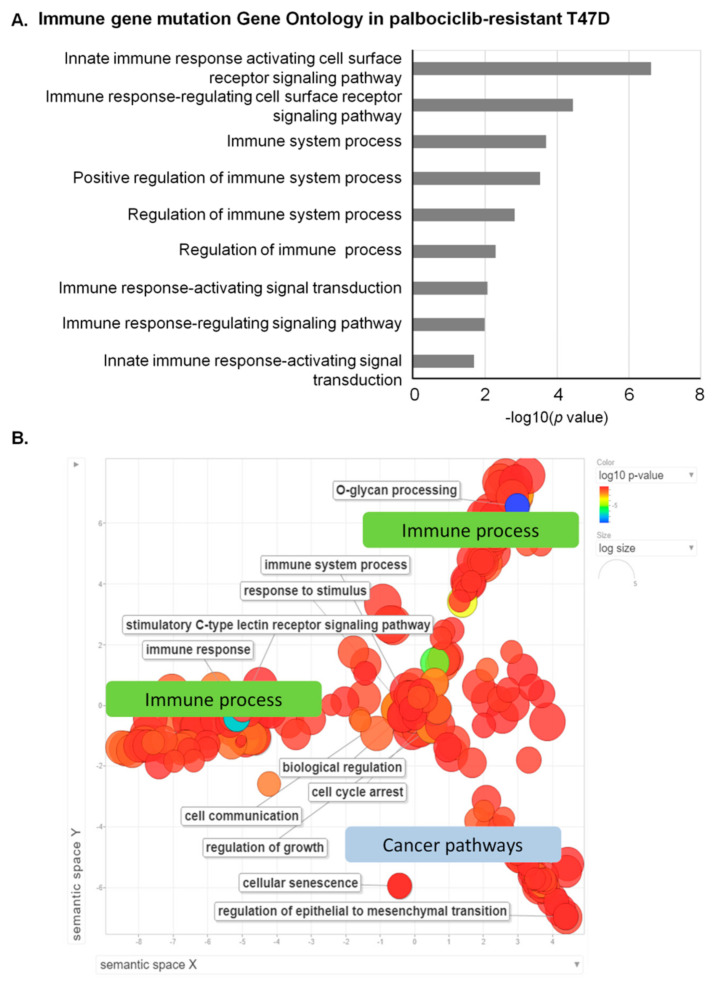
Mutation profiling in T47D or T47D-PR cells revealing immune pathway deregulation in palbociclib-resistant cells. (**A**) Mutations were identified according to the Genome Analysis Toolkit v3.4.0 following their best practice guidelines as described in the Methods section. The bar graph shows that GO Biological Process terms related to immune response were enriched in genes with mutations occurring in T47D or T47D-PR cells. The X axis indicates *p* values in the specified formula −log 10 (*p* value). The bar represents the statistical significance of each GO term. All GO terms in Figure 3 were statistically significant (adjusted *p* value < 0.25). The gene list of each GO term overlapping with genes with mutations is provided in Table 2. (**B**) Visualization of GO enrichment in mutated genes from T47D or T47D-PR cells revealed prominent immune process involvement in palbociclib-resistant cells. The scatterplot of representative GO terms shows the relationship between significantly enriched GO terms and genes with driver or pathogenic mutations occurring in T47D cells. The color of the circle indicates the statistical significance of the GO term in log10 of the *p* value. The most significant GO terms are shown in blue, while those with the lowest significance are presented in red. The size of the circle indicates the frequency of the GO term in the GO database, with more general GO terms having a larger size. There are three clusters in the semantic space of the scatterplot. The two clusters in the upper part are related to the immune process, while the other one in the lower part is related to cancer pathways.

Based on WES data, we identified 203 clinically important mutations in 107 genes from T47D or T47D-PR cells (Supplementary Table S1 and Figure S2) and 312 mutations in 150 genes from MCF7 or MCF7-PR cells (Supplementary Table S2 and Figure S3). Although no GO terms were significantly enriched in the 150 genes from MCF7 or MCF7-PR cells (adjusted *p* value > 0.25) (Supplementary Figure S4A and Table 1), several immune-related GO terms were significantly enriched in the 107 genes from T47D or T47D-PR cells (adjusted *p* value < 0.25) (Figure 3A, Supplementary Figure S4B and Table 2). In particular, surface mucin genes, which play important roles in protecting epithelial cells and have been implicated in epithelial renewal and differentiation [31], were highly mutated in T47D-PR cells (Table 2). These mucin genes were also reported to be involved in immune regulation [32,33].

### 3.3. Visualization of GO Revealing Prominent Immune Process Involvement in Palbociclib-Resistant Cells

GO enriched terms in the 107 genes with mutations in T47D or T47D-PR cells (Supplementary Figure S4B and Table S2) were analyzed and visualized using REVIGO [27] (Figure 3B). For a better understanding of enriched GO terms, REVIGO measures the relationship between GO terms, removes redundant terms based on similarity scores, and intuitively visualizes the representative GO term sets. When visualizing enriched GO terms, three clusters were observed. Accordingly, two clusters in the upper part of the semantic space of Figure 3B were related to immune pathway, such as immune response and response to stimulus, while one cluster in the lower part was related to the cancer pathway, such as cell cycle arrest, cellular senescence, and regulation of epithelial to mesenchymal transition.

### 3.4. Mutation Dot Plots

**Figure 4:**
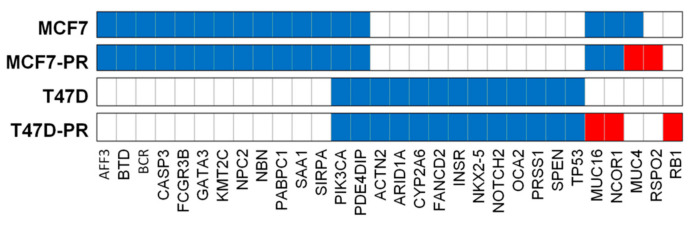
Mutation dot plot of 30 genes with 52 of the most clinically significant mutations. We selected mutations with only High impact or pathogenic mutations in ClinVar for the mutation dot plot. Mutations of the parent cells that remained in the resistant cells are colored blue, while newly exhibited mutations in the resistant cells are colored red. The bar graph represents the number of mutations in each cell. T47D-PR cells obtained the following new mutations: *MUC16* (K13558fs)*, NCOR1* (R190*), and *RB1* (Y659fs). Meanwhile, MCF7-PR cells exhibited the following new mutations: *MUC4* (M3855fs, L3857fs, T3860fs, P3862fs) and *RSPO2* (R64*).

We herein identified 52 of the most clinically significant mutations in 30 genes as described in the Methods section (Supplementary Table S3, Figure 4, and Supplementary Figure S5). As shown in Figure 4, MCF7-PR cells retained 29 mutations in 16 genes from MCF7 cells, among which six (*BTD* (G47R), *FCGR3B* (I142V), *NBN* (R43*), *NPC2* (c.441+1G), *PIK3CA* (E545K), and *SAA1* (A70V)) were reported as pathogenic in ClinVar, (National Center for Biotechnology Information) [34]. T47D-PR cells also retained 16 mutations in 13 genes from T47D cells, among which nine *(ACTN* (T716M), *CYP2A6* (L160H), *INSR* (V1012M), *NKX2-5* (E21Q), *OCA2* (A481T), *PIK3CA* (H1047R), *PRSS1* (A16V) and (N54S), and *TP53* (L194F)) were pathogenic in ClinVar.

Both MCF7 and T47D cells and their palbociclib-resistant variants (MCF7-PR and T47D-PR) had mutations in *PDE4DIP* and *PIK3CA*. Interestingly, T47D-PR cells exhibited *NCOR1* (R190*), *MUC16* (K13558fs), and *RB1* (Y659fs) mutations not found in T47D cells. In addition, MCF7-PR cells exhibited mutations in *MUC4* (M3855fs, L3857fs, T3860fs, P3862fs) and *RSPO2* (R64*), which were not found in MCF7 cells. The association between mutated genes found only in palbociclib-resistant cells and CDK4/6 inhibitor resistance warrants further investigation. Among the four mutated genes described above (i.e., *NCOR1, MUC16, MUC4,* and *RSPO2)*, *NCOR1, MUC16,* and *MUC4* were reported to have been directly involved in modulating tumor microenvironment and thereby mediating drug resistance [35,36,37]. Whereas, *RSPO2* is indirectly correlated to affect immune response by activating Wnt/beta-catenin signaling regulating T cell-inflammation in the tumor microenvironment [38,39]. In terms of mutation frequency, of a total of 58 mutated genes in T47D-PR cells, eight genes are mucin genes (13.8%). Those eight mucin genes have 30 mutations (30.0%) and another 50 genes have 70 mutations. Therefore, the mucin genes have significantly more frequent mutations than other genes (*p* = 0.022).

## 4. Discussion

Little is known regarding the association between CDK4/6 inhibitor resistance and immune pathways. Using our own palbociclib-resistant preclinical model, the present study demonstrated that palbociclib resistance was associated with a deregulated immune pathway. Our findings may help guide future research regarding immune pathway regulation to overcome CDK4/6 inhibitor resistance. 

To identify alterations that promote palbociclib resistance, we generated palbociclib-resistant preclinical models using two different HR-positive cell lines harboring different genomic profiles including hotspot mutations in the *PIK3CA* gene (i.e., exon 9 (E545K) in MCF7, exon 20 (H1047R) in T47D) [40] and variations in *P53* gene (i.e., *P53* wildtype in MCF7, mutated in T47D). This may reflect genomic heterogeneity among patients. Even though bystander genomic changes might occur in these cells overtime during long-term cultivation, we compared those long-term palbociclib-exposed cells with the non-treated fresh cells to mimic paired pretreatment and posttreatment clinical samples in clinical settings for the genomic characterization of acquired drug resistance [41].

Regarding gene expression analyses associated with CDK4/6 inhibitor resistance, only CCNE1 (cyclin E) overexpression [42] and RB loss [43,44] were clinically confirmed in the literatures. In our resistant model, we also observed CCNE overexpression i.e., activation of cyclinE-CDK2 pathway and RB loss associated with palbociclib resistance [20]. Additionally, in search of other possible pathways that could be responsible for the development of resistance to CDK4/6 inhibitor, we analyzed genomics and transcriptomics of palbociclib-sensitive and resistant breast cancer cells. When we analyzed enriched GO pathway in DEGs, the most noticeable pathways were immune-related ones. Except immune pathways, there were several cancer pathways related to cell death, apoptosis and proliferation. However, since they were all related to general cancer biological process, they could not suggest the molecular relevance for acquired palbociclib-resistance mechanisms. Therefore, we focused on immune pathway genes which may be associated with CDK4/6 inhibitor resistance.

Studies have reported that IFN signaling activation was correlated with cancer progression and emergence of drug resistance [45]. IFNs have been known to activate the JAK/STAT pathway, which promotes tumorigenesis and drug resistance via enhancement of epithelial–mesenchymal transition [45]. Mounting evidence has also indicated that genes involved in type 1 IFN signaling are involved in endocrine resistance [28,46]. Moreover, recent reports have revealed enrichment of type 1 IFN signaling and induction of IL6/STAT3 pathway in CDK4/6 inhibitor-resistant cells [29,47]. Similarly, the current study supported the previous results by demonstrating that a panel of type 1 IFN pathway genes, including *SP100, IRF9*, and *STAT1*, was overexpressed in palbociclib-resistant cells. This warrants further investigation on the type I IFN pathway as a potential diagnostic or therapeutic target for CDK4/6 inhibitor-resistant patients. Based on the preclinical evidences that CDK4/6 inhibition augments immune response [18,19] as mentioned earlier, CDK4/6 inhibitor and immunotherapy combinations have currently been evaluated in a couple clinical trials [48,49]. Preliminary results of this phase Ib trial demonstrated that the combination of abemaciclib and pembrolizumab was safe and exhibited promising in antitumor activity [48]. This prompted us to investigate alterations in immune checkpoint genes in our palbociclib-resistant preclinical model. Interestingly, we newly found that while immune checkpoint inhibitory genes increased, immune checkpoint stimulatory genes decreased in our palbociclib-resistant preclinical model. This association between CDK4/6 inhibitor resistance and altered immune checkpoint pathway has never been previously reported. Future studies are therefore needed to obtain further insight into the combined use of CDK4/6 inhibitors and immunotherapy to overcome CDK4/6 inhibitor resistance.

We have compared our WES results to the literature where 59 clinical samples obtained pre- and post-CDK4/6 inhibitor treatment were analyzed by WES [43]. In that literature, mutations associated with intrinsic resistance to CDK4/6 inhibitor were in *RB1, AKT1, KRAS, FGFR2, ERBB2, CCNE2, AURKA,* and *ER* genes and mutations associated with acquired resistance were in *RB1* and *AKT1* genes. On the other hand, what we found in palbociclib-resistant cells were mutations in *RB1, NCOR1, MUC4, MUC16*, and *RSPO2* genes. Of these, *NCOR1, MUC4*, and *MUC16* genes have been investigated for their aberrant expression in various cancers and being attractive targets for immunotherapy in previous studies [37,50,51]. As mentioned earlier, since mucin genes were more frequently mutated than other genes, mucin genes may draw attention from researchers. Aberrant glycosylation of mucins interferes with the ability of natural killer cells to destroy tumor cells, suggesting immunosuppression around the tumor [33]. In addition, altered expression and glycolysation of mucin have been reported to hinder the activation of cytotoxic T-lymphocyte thereby enabling cancer cell survival [52]. On the other hand, *NCOR1* has been found to have an important function in proper T cell development [53]. Moreover, downregulation of *NCOR1* in dendritic cells has been reported to induce FoxP3+ regulatory T cells by inhibiting Th17 cell development [37]. However, the association between mutations in such genes and CDK4/6 inhibitor resistance observed herein has never been documented in both the clinical samples and preclinical models thus far. Our novel mutations in *NCOR1, MUC4,* and *MUC16* genes should be validated in further mechanistic studies and translational studies so that those mutations might serve as predictive biomarkers or therapeutic targets for patients with CDK4/6 inhibitor-resistant breast cancer.

Our study has some limitations. Validation of biological functions of all the identified genes is lacking here. Despite that, our initial genomic study will help guide further research to validate the association of immune pathway in driving palbociclib resistance. In addition, we acknowledge that this model might not fully satisfy the clinical scenario where resistance evolves in combined endocrine-palbociclib treatment setting. However, here we generated solely palbociclib resistant models in order to identify mechanisms specifically related to palbociclib resistance. Another project of ours is underway to reveal resistance to combined fulvestrant and palbociclib using a preclinical animal model of resistance to both drugs.

## 5. Conclusions

In conclusion, after screening and analyzing various genes and their respective pathways, the present study was able to determine the deregulated immune pathway as an additional mechanism of CDK4/6 inhibitor resistance besides the activation of cyclin E-CDK2 pathway and loss of RB etc. Although our finding requires further validation, in the era of anticancer immunotherapy, immune associated resistance mechanisms such as PD-1/PD-L1 activation could be of great interest and might potentially be targeted with immune checkpoint inhibitors. Therefore, potential association of CDK4/6 inhibitor resistance and immune pathway warrants vigorous further research.

## Figures and Tables

**Table 1:** GO Biological Process terms related to immune responses were enriched in DEGs between MCF7-PR and MCF7 cells.

GO Biological Process	Count	Genes	*p*-Value	Adjusted *p*-Value
immune response	54	*ADCY5, ANG, ANXA3, BLNK, BST2, CD22, CEBPG, CLEC2D, CLU, CTSC, CTSH, CTSK, DDX58, DDX60, EGR1, EPRS, FFAR3, FRK, FTH1, GBP2, HERC5, HMOX1, IFI6, IFIH1, IFIT1, IFITM1, IGHD, IL20, IRF9, ISG15, KIR2DS2, KIR2DS4, KIR3DL2, KYNU, LYN, MYB, NFIL3, OAS1, OAS2, OAS3, PTGER4, RAET1G, RIPK2, S100A8, S100A9, SEMA3C, STAT1, SUSD2, TAB1, TXNIP, ULBP1, UNC13D, USP18, VIPR1*	1.5 × 10^−10^	4.3 × 10^−9^
immune effector process	33	*ACKR3, ANXA3, BST2, CEBPG, CLU, CTSC, CTSH, DDX58, DDX60, FFAR3, HERC5, HMOX1, IFIH1, IFIT1, IFITM1, IGHD, IRF9, ISG15, LYN, MTSS1, MYB, OAS1, OAS2, OAS3, PDK4, PTGER4, RAET1G, RIPK2, STAT1, TNIK, ULBP1, UNC13D, ZNF189*	2.7 × 10^−9^	6.8 × 10^−8^
regulation of immune system process	45	*ACKR3, ANXA1, BMP4, BST2, C5AR2, CDK6, CLEC2D, CLU, CTSH, CTSK, DDX58, DDX60, FFAR3, FLT3, HERC5, HMOX1, IFIH1, IFIT1, IFITM1, IGHD, IL20, ISG15, KIR2DS2, KIR3DL2, LYN, MITF, MTSS1, MYB, MYC, PDE5A, PDK4, PLCB1, PTGER4, RIPK2, S100A7, SHPK, STAT1, TAB1, TGFBR2, TNIK, TRIB1, ULBP1, UNC13D, USP18, ZNF189*	1.4 × 10^−7^	2.8 × 10^−6^
innate immune response	36	*ADCY5, ANG, BST2, CEBPG, CLU, CTSK, DDX58, DDX60, EGR1, EPRS, FRK, GBP2, HERC5, IFI6, IFIH1, IFIT1, IFITM1, IRF9, ISG15, KIR2DS2, KIR2DS4, KYNU, LYN, OAS1, OAS2, OAS3, RAET1G, RIPK2, S100A8, S100A9, STAT1, TAB1, TXNIP, ULBP1, UNC13D, USP18*	3.5 × 10^−7^	6.5 × 10^−6^
immune system development	30	*ANXA1, BMP4, CALCR, CDK6, CEBPG, DHRS2, EGR1, FLT3, G6PD, HERC6, IGHD, IL20, ISG15, KRT75, L3MBTL3, LYN, MITF, MPZL2, MYB, MYC, ONECUT1, PTGER4, RIPK2, RUNX2, SIX1, TGFBR2, TMOD2, TRIB1, ZFP36L2, ZNF385A*	4.5 × 10^−7^	8.1 × 10^−6^
regulation of immune effector process	16	*ACKR3, BST2, DDX58, DDX60, FFAR3, HERC5, HMOX1, IFIT1, LYN, MTSS1, MYB, PDK4, RIPK2, TNIK, UNC13D, ZNF189*	0.00018	0.00182
negative regulation of immune system process	13	*BMP4, BST2, C5AR2, CDK6, FLT3, HMOX1, IFIT1, LYN, MYC, PDE5A, PLCB1, PTGER4, TRIB1*	0.00155	0.01199

**Table 2:** GO Biological Process terms related to immune responses were enriched in genes with mutations occurred in T47D cells.

GO Biological Process	Count	Genes	*p*-Value	Adjusted *p*-Value
innate immune response activating cell surface receptor signaling pathway	7	*MUC3A, MUC4, MUC5B, MUC6, MUC12, MUC16, MUC17*	2.41 × 10^−7^	7.24 × 10^−5^
immune response-regulating cell surface receptor signaling pathway	8	*HSP90AB1, MUC3A, MUC4, MUC5B, MUC6, MUC12, MUC17, SOS1*	3.62 × 10^−5^	0.0048
innate immune response-activating signal transduction	6	*MUC3A, MUC4, MUC5B, MUC6, MUC12, MUC17*	0.0002	0.0238
immune response-regulating signaling pathway	8	*HSP90AB1, MUC3A, MUC4, MUC5B, MUC6, MUC12, MUC17, SOS1*	0.0003	0.0238
immune response-activating signal transduction	7	*HSP90AB1, MUC3A, MUC4, MUC5B, MUC6, MUC17, MUC12*	0.0015	0.0895
regulation of immune response	8	*HSP90AB1, MUC3A, MUC4, MUC5B, MUC6, MUC17, MUC12, SOS1*	0.0052	0.1476
regulation of immune system process	11	*HSP90AB1, KMT2C, MUC3A, MUC4, MUC5B, MUC6, MUC12, MUC17, POU4F1, RB1, SOS1*	0.0086	0.1476
positive regulation of immune system process	9	*HSP90AB1, MUC3A, MUC4, MUC5B, MUC6, MUC12, MUC17, POU4F1, RB1*	0.0103	0.1476
immune system process	12	*PRSS3, KMT2C, HSP90AB1, TGFBR1, SOS1, MUC5B, MUC4,* *VCP, MUC17, MUC3A, MUC6, MUC12*	0.0203	0.1476

## Supplementary Materials

The following are available online at https://www.mdpi.com/2073-4425/12/2/159/s1, Figure S1: Bar graphs showing the enrichment of general GO biological process terms in DEGs in both parent and resistant cells, Figure S2: Venn diagram showing the distribution of the 107 genes with clinically significant mutations from T47D or T47D-PR cells, Figure S3: Venn diagram showing the distribution of the 150 genes with clinically significant mutations from MCF7 or MCF7-PR cells, Figure S4: Bar graphs showing the enrichment of general GO biological process terms in genes with driver or pathogenic mutations from all four cells, Figure S5: Venn diagram showing the distribution of the 30 genes with 52 of the most clinically significant mutations from MCF7 or MCF7-PR cells and T47D or T47D-PR cells. Table S1: Summary of the 203 clinically significant mutations in 107 genes from the T47D cells based on whole-exome sequencing data, Table S2: Summary of the 312 clinically significant mutations in 150 genes from the MCF7 cells based on whole-exome sequencing data, Table S3: Summary of the 52 most clinically significant mutations in 30 genes, Table S4: GO Biological Process terms related to non-immune pathways were enriched in DEGs between MCF7-PR and MCF7 cell line.
