# Supplementary material for: Deregulated Immune Pathway Associated with Palbociclib Resistance in Preclinical Breast Cancer Models: Integrative Genomics and Transcriptomics
Source: Genes (Basel). 2021 Jan 25;12(2):159. doi: 10.3390/genes12020159 (PMC7912104; doi:10.3390/genes12020159)
Supplement: Supplementary file 1 [file genes-12-00159-s001.zip › genes - 1049728 - supplementary/supplementary figures.docx]

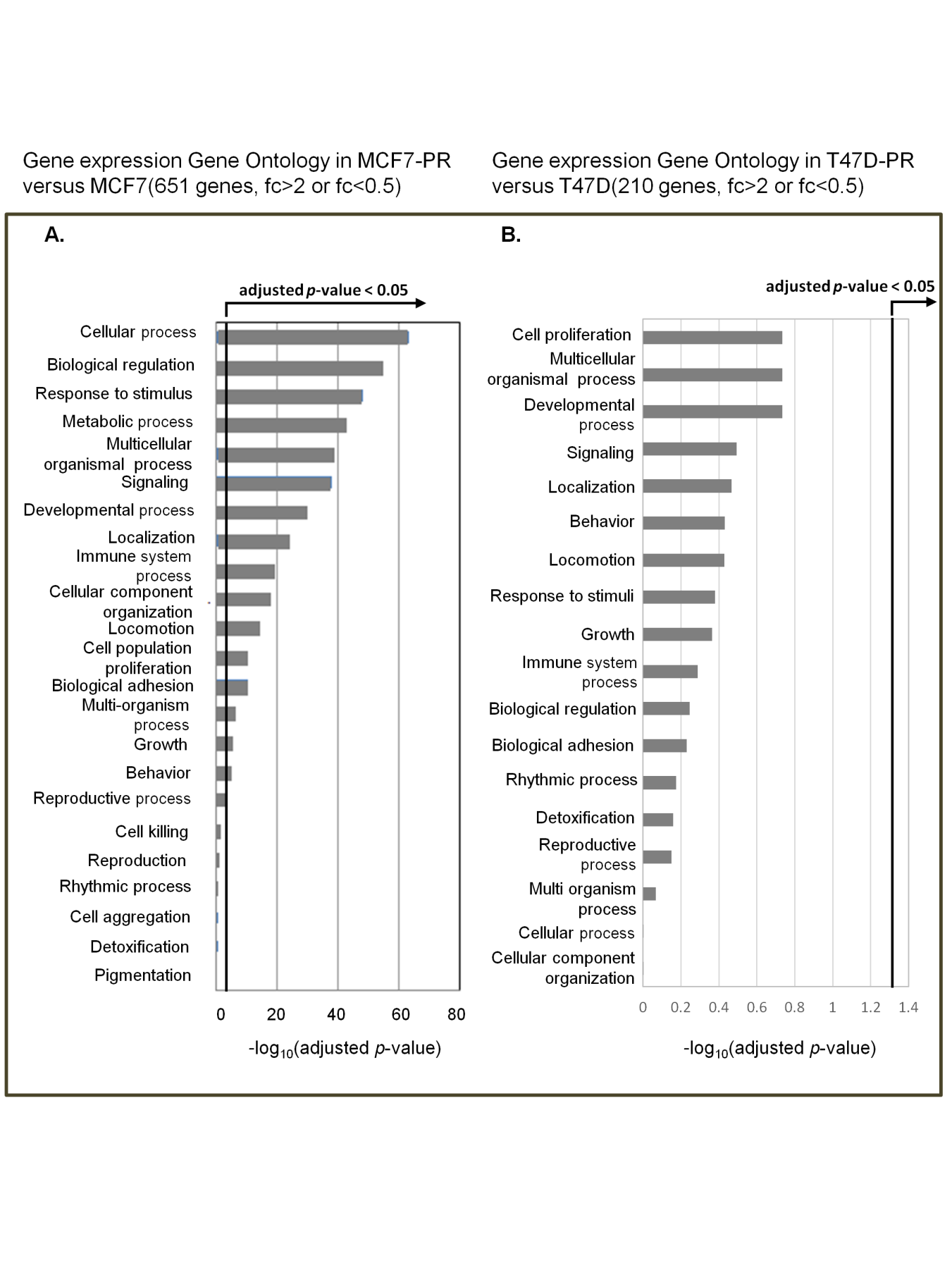
 **
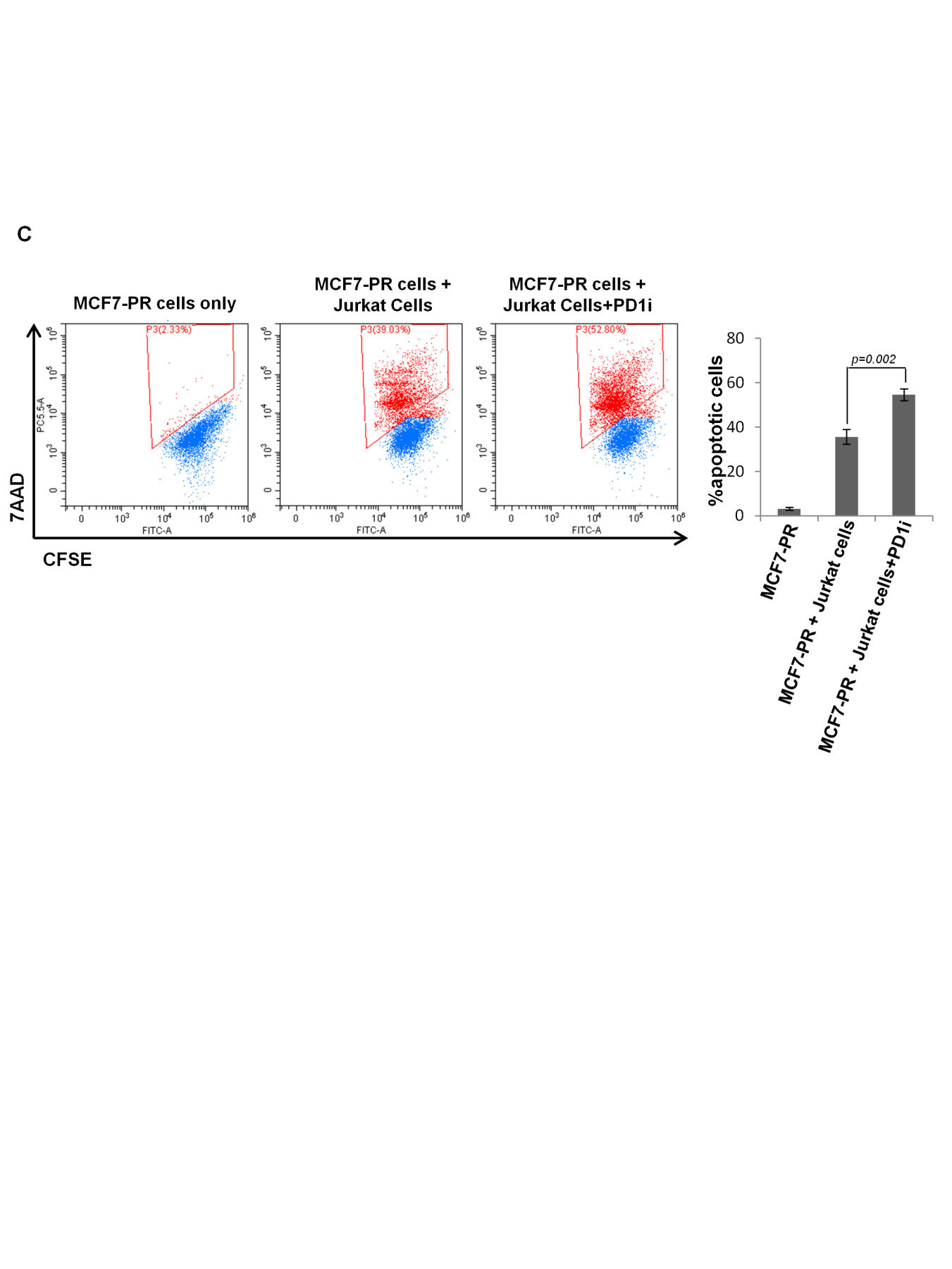
**

**Supplementary Figure 1(A-B).** Bar graphs showing the enrichment of general GO biological process terms in DEGs in both parent and resistant cells. Given that the GO terms presented in this figure are the first children terms of the biological process, they show which general GO terms were enriched in those DEGs. (A) The bar graph shows the enrichment of the GO terms in DEGs from the MCF7 and MCF7-PR cells. The bar represents the statistical significance of each GO term. Many biological process terms, such as cellular process and response to stimulus, including the immune system process, were enriched in DEGs from the MCF7 cells. (B) Panel B shows that the enrichment of GO terms in DEGs from the T47D and T47D-PR cells. No GO terms were enriched in DEGs from the T47D cells. **(C)** Cell-mediated cytotoxicity assay demonstrating the increased cancer cell killing activity of Jurkat cells, when treated with PD-1 inhibitor compared to Jurkat cells co-culture with MCF7-PR cells only. *p*-values were calculated by student’s t-test. Data are presented as the mean ± S.E.M. of triplicate experiments.

**
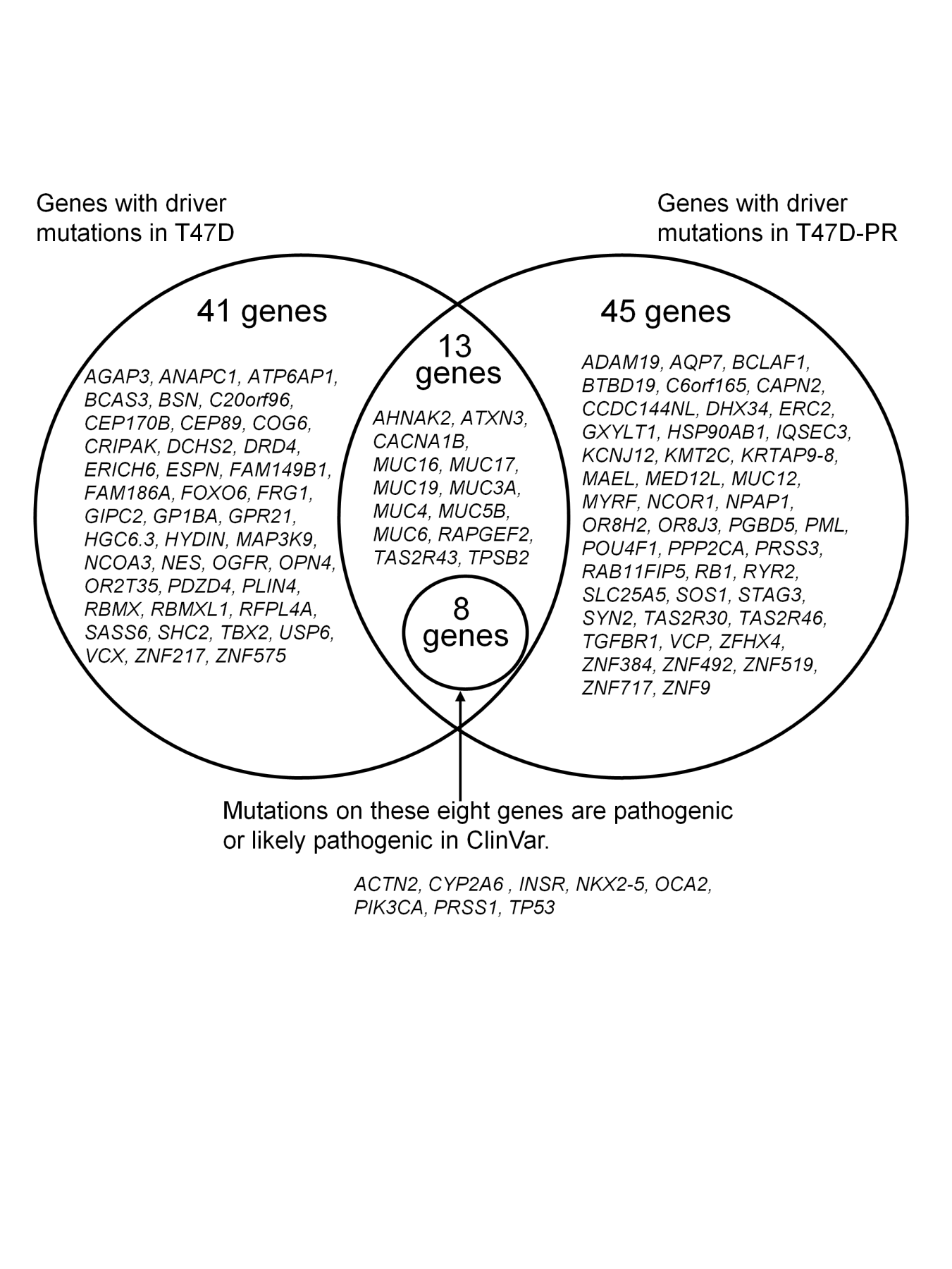
**

**Supplementary Figure 2.** Venn diagram showing the distribution of the 107 genes with clinically significant mutations from T47D or T47D-PR cells. Among the 107 genes, 62 and 66 had mutations in T47D and T47D-PR cells, respectively. A total of 21 genes from both T47D and T47D-PR cells overlapped, among which 8 were found to have pathogenic mutations in ClinVar.

**
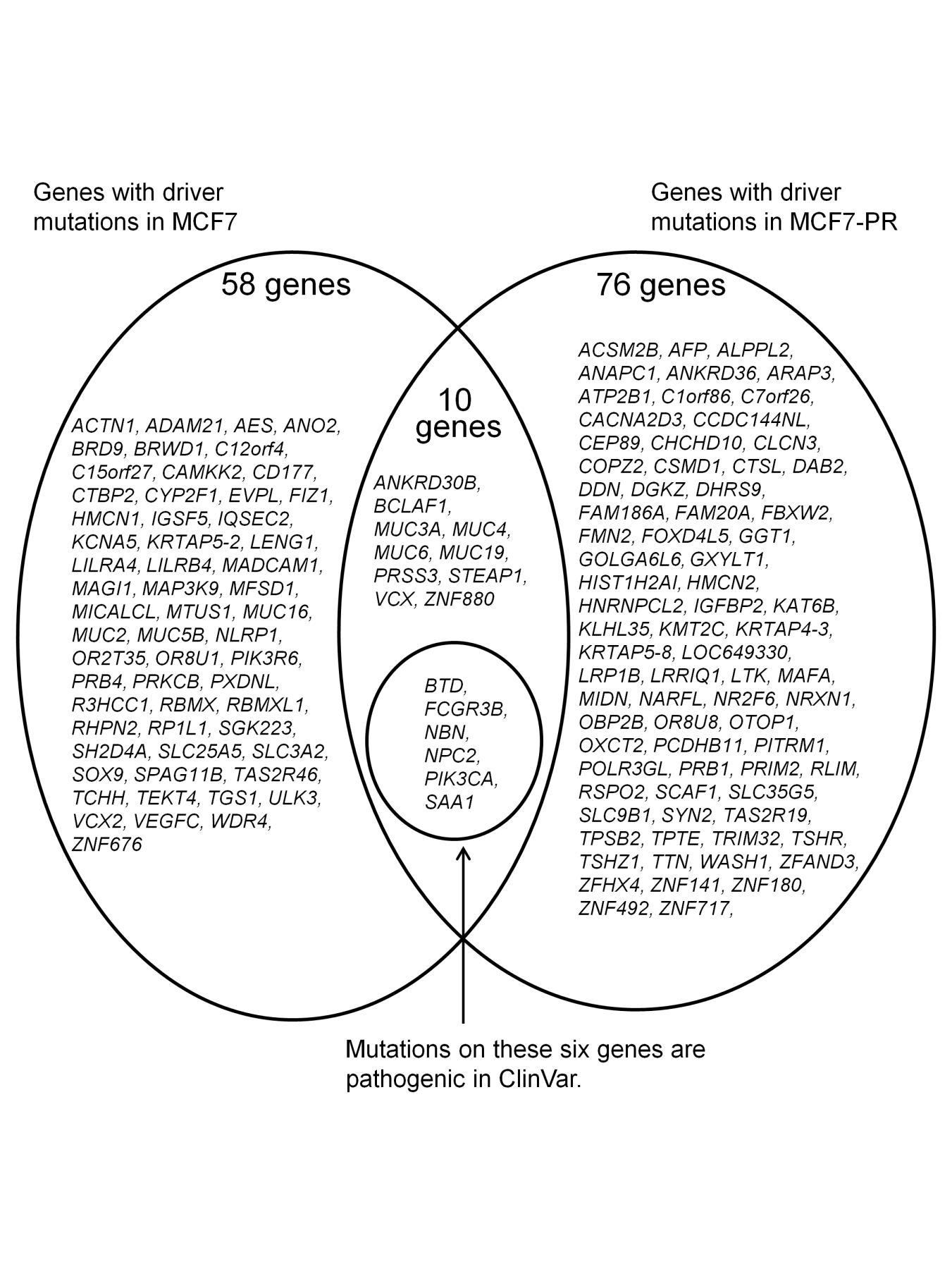
**

**Supplementary Figure 3.** Venn diagram showing the distribution of the 150 genes with clinically significant mutations from MCF7 or MCF7-PR cells. Among the 150 genes, 74 and 92 had mutations in MCF7 and MCF7-PR cells, respectively. A total of 16 genes from both MCF7 and MCF7-PR cells overlapped, among which six were found to have pathogenic mutations in ClinVar.


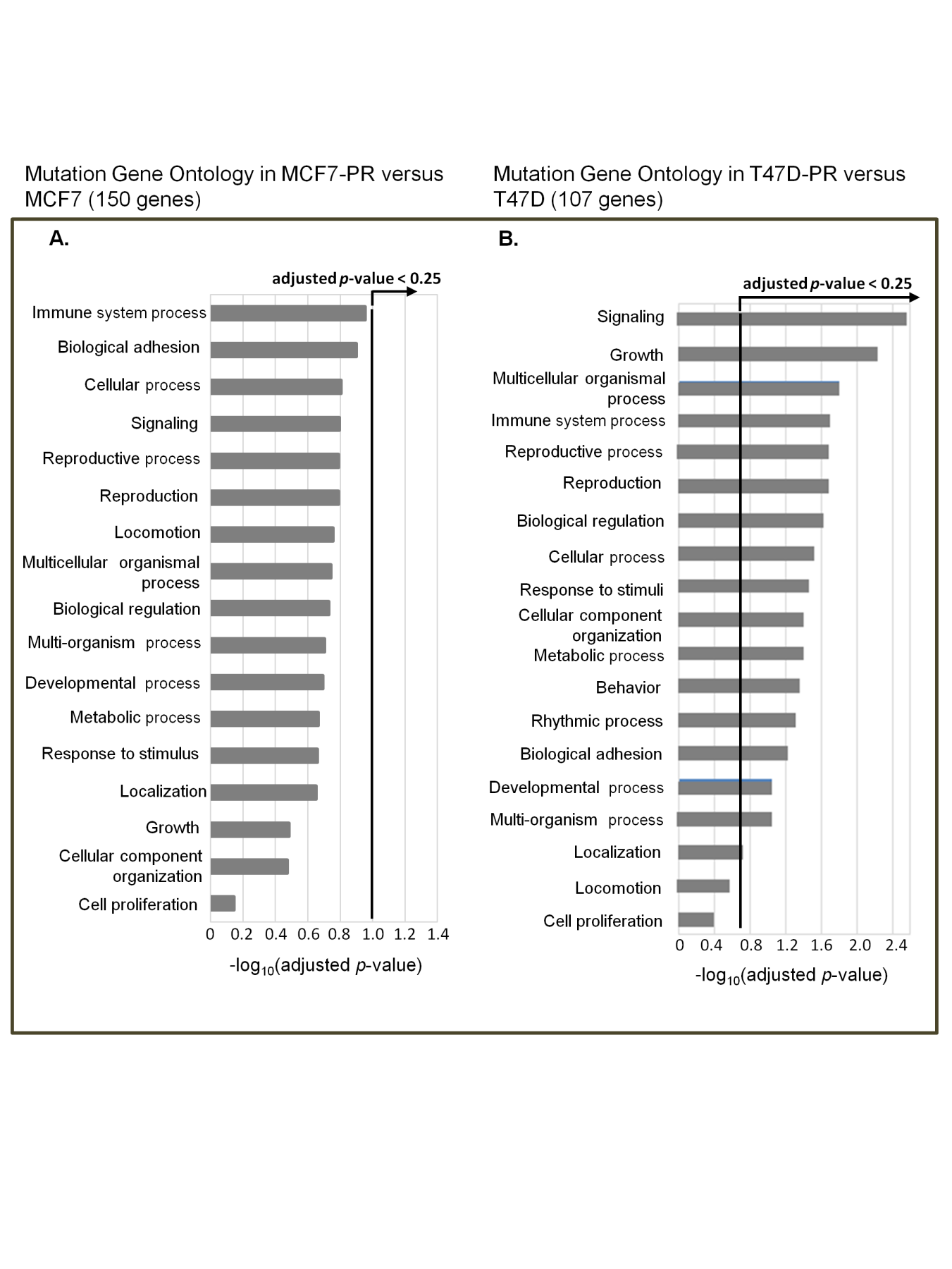


**Supplementary Figure 4.** Bar graphs showing the enrichment of general GO biological process terms in genes with driver or pathogenic mutations from all four cells. (A) The bar graph shows the enrichment of GO terms in genes with driver or pathogenic mutations from MCF7 or MCF7-PR cells. No GO terms were enriched in mutated genes from MCF7 or MCF7-PR cells. (B) Panel B shows the enrichment of GO terms in genes with driver or pathogenic mutations from T47D or T47D-PR cells. Several GO terms, such as signaling and growth, including the immune system process, were enriched in mutated genes from T47D or T47D-PR cells.

**
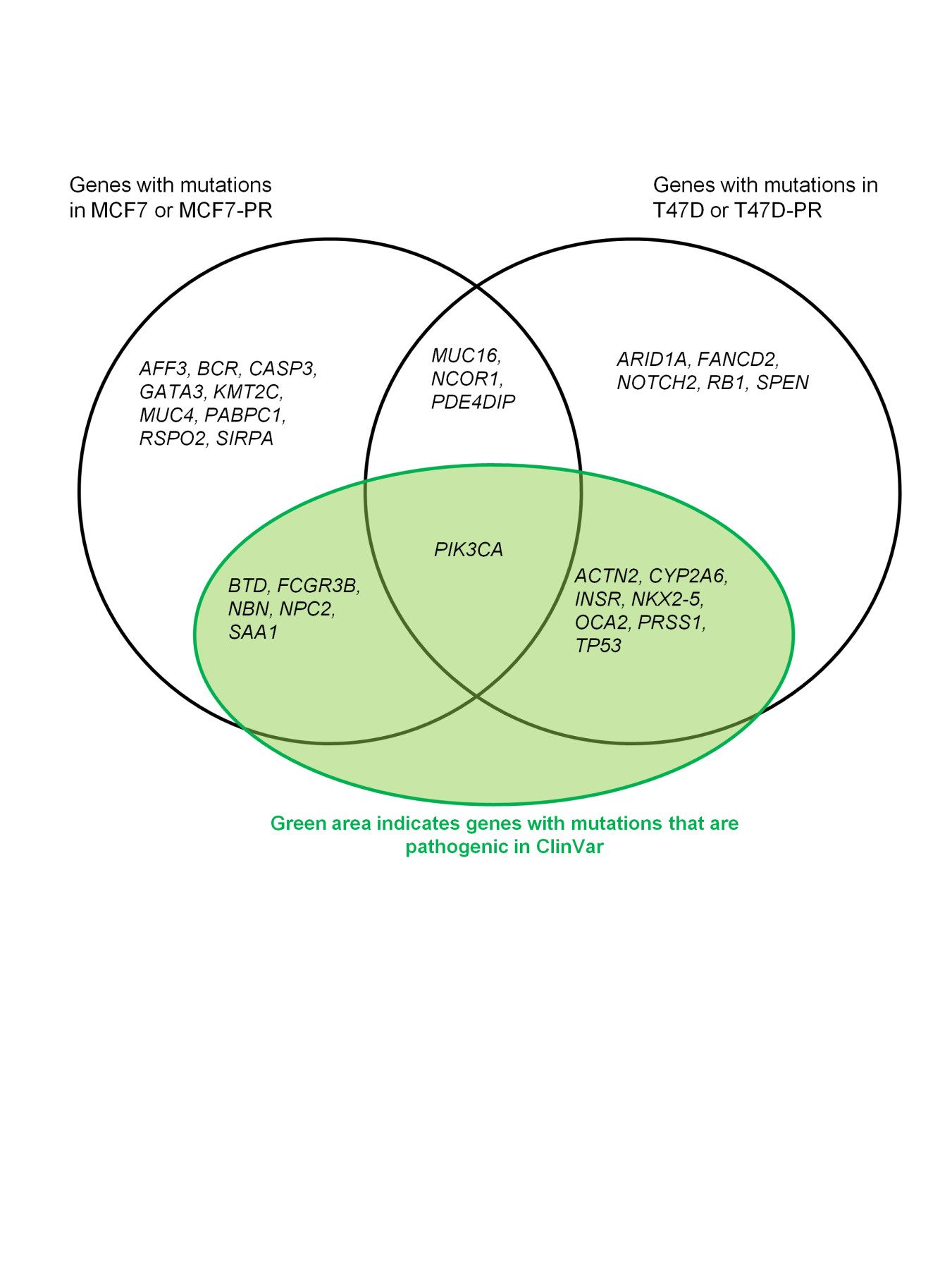
**

**Supplementary Figure 5.** Venn diagram showing the distribution of the 30 genes with 52 of the most clinically significant mutations from MCF7 or MCF7-PR cells and T47D or T47D-PR cells. Genes encircled with a green line have mutations that are pathogenic in ClinVar.
